# Microalgae a Superior Source of Folates: Quantification of Folates in Halophile Microalgae by Stable Isotope Dilution Assay

**DOI:** 10.3389/fbioe.2019.00481

**Published:** 2020-01-21

**Authors:** Dirk Volker Woortman, Tobias Fuchs, Lisa Striegel, Monika Fuchs, Nadine Weber, Thomas B. Brück, Michael Rychlik

**Affiliations:** ^1^Werner Siemens-Chair of Synthetic Biotechnology, Technical University of Munich, Garching, Germany; ^2^Chair of Analytical Food Chemistry, Technical University of Munich, Freising, Germany; ^3^Centre for Nutrition and Food Sciences, Queensland Alliance for Agriculture and Food Innovation, The University of Queensland, Brisbane, QLD, Australia

**Keywords:** stable isotope dilution assay, microalgae, folates, *Chlorella*, *Dunaliella*, *Picochlorum*

## Abstract

A multitude of human nutritional supplements based on *Chlorella vulgaris* biomass has recently been introduced to the specialty food market. In this study, an analysis of total folate contents in *Chlorella* sp. and a series of marine microalgae was conducted to evaluate folate content in alternative algae-based food production strains. For the first time, total folate content and vitamer distribution in microalgae were analyzed by stable isotope dilution assay (SIDA) using LC-MS/MS, which has demonstrated its superiority with respect to folate quantification. Consistently, high folate contents were detected in all examined microalgae samples. High folate concentrations of 3,460 ± 134 μg/100 g dry biomass were detected in freshly cultivated *Chlorella vulgaris*, notably also in other well-researched microalgae strains. To that end, the highest folate content currently documented for any algae sample was measured in the marine microalgae *Picochlorum* sp. isolate with values of 6,470 ± 167 μg/100 g dry biomass. This calls for alternative products based on other algae biomass. Our data indicate that freshwater and marine microalgae provide extremely high concentrations of folates, which warrant further studies on the regulation of pteroylpolyglutamates in algae as well as on bioaccessibility, absorption, and retention in humans.

## Introduction

With an ever-growing global population and increasing limiting availability of agricultural land, there is a growing demand for edible biomass that contains high concentrations of macro- and micronutrients (Tilman et al., 2002; Foley et al., 2011; Lipper et al., 2014). This situation is further aggravated by climate change effects, which ultimately lead to a shift and total reduction of agricultural lands (Tilman et al., 2001). Hence, generating nutrient concentrated, edible biomass on non-agricultural landmass is a pivotal role for a food-centered, future biorefinery approach (Laurens et al., 2017; Sheppard et al., 2019). To that end, microalgae potentially yield at least five times more biomass than terrestrial plants on the equivalent land surface and can be cultivated without the need for freshwater (Benedetti et al., 2018). Therefore, microalgae cultivation does not compete with terrestrial agricultural activity but can act synergistically to generate concentrated food resources (Vanthoor-Koopmans et al., 2013). Hence, generating a microalgae-based biorefinery that generates various performance nutrient outlets, such as proteins, sugars and polyunsaturated fatty acids can significantly contribute to meet food demands of future generations (Subhadra and Grinson-George, 2011). While microalgae-based production of macronutrients such as sugars and proteins has been elucidated extensively in the literature, detailed studies on micronutrient, such as vitamins, which add significant economic and nutritional value to food-centered microalgae biorefinery, have been limited (Pulz and Gross, 2004; Draaisma et al., 2013; van der Spiegel et al., 2013). In that respect, Vitamin B9 (Folate) is of extensive importance due to its function in human development and health maintenance. The marine microalgae have recently been flagged as concentrated food production platforms for human and animal nutrition due to their high concentration of health-promoting nutrients, such as proteins and polyunsaturated fatty acids (PUFAs) (Harun et al., 2010; Grosso et al., 2014; Kent et al., 2015; Marventano et al., 2015). It has been reported that high vitamin containing algae biomass could be used to address malnutrition in populations at risk, which not only encompass pregnant women with higher nutritional demands, but also poverty-stricken populations (Pratt and Johnson, 1965; Brown et al., 1999; Becker, 2003; Christaki et al., 2011). A multitude of human nutritional supplements based on algae biomass has recently been introduced. Nevertheless, the often-cited nutritional value of algae food supplements with respect to their micronutrient content and bioavailability is still a matter of scientific debate (Brown et al., 1999; Wells et al., 2017).

**Figure 1:**
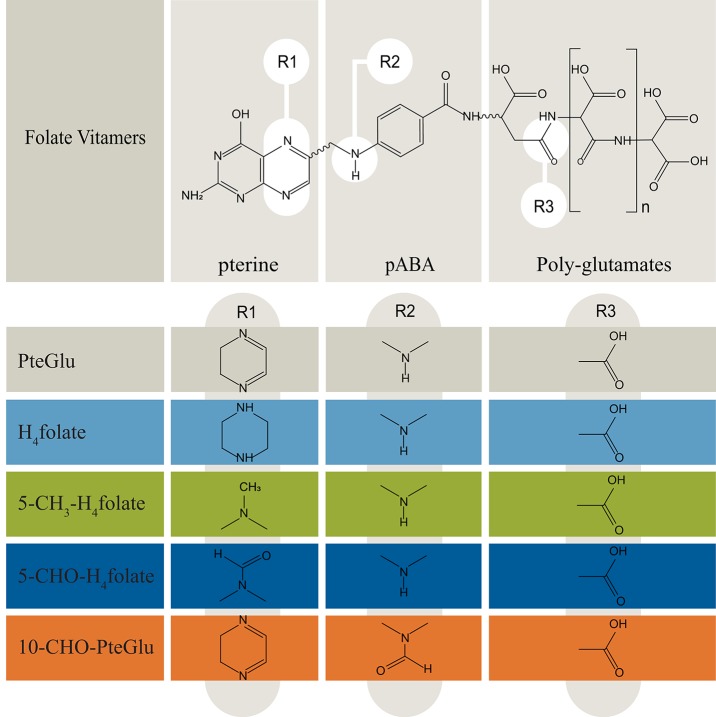
Overview of folate vitamers chemistry (Delchier et al., 2016). Folates are mostly present in the polyglutamated form, which need to be cleaved during sample preparation for quantitative LC-HRMS measurements. pABA, para-aminobenzoic acid. *n*, number of glutamates: 1–8.

Folate vitamins are essential to human nutrition and necessary for many one carbon metabolic pathways, particularly in the synthesis of amino acids and nucleotides (Shane, 1989). A deficiency of folates in women before and during pregnancy is related to an increased prevalence of neural tube defects (NTDs) in newborns. In contrast to humans, plants synthesize folates *de novo* and, therefore, provide dietary folates for humans. These compounds are accruing in all cellular compartments, such as mitochondria, cytoplasm, and nucleus, where they carry out distinct biochemical functions (Selhub, 2002). Folates consist of three different chemical building blocks, i.e., pterin, para-amino benzoic acid (pABA), and varying numbers of glutamate residues. Depending on the oxidation state, various C1 substituents, and the polyglutamyl tail lengths, the most abundant forms in plants are H_4_folate (tetrahydrofolic acid; 2-((4-((2-Amino-4-oxo-5,6,7,8-tetrahydro-1H-pteridin-6-yl)methylamino)benzoyl)-amino)pentandisäure), 5-CH_3_-H_4_folate (5-methyltetrahydrofolate; (2S)-2-[[4-[(2-Amino-5-methyl-4-oxo-1,6,7,8-tetrahydropteridin-6-yl) methylamino]benzoyl]amino]pentanedioic acid), 5-CHO-H_4_folate (*5*-formyltetrahydrofolate, 2-[[4-[(2-amino-5-formyl-4-oxo-3,6,7,8-tetrahydropteridin-6-yl)methylamino]benzoyl]amino]pentanedioic acid), 10-CHO-PteGlu (10-formyl-folate (2S)-2-[[4-[(2-amino-4-oxo-3H-pteridin-6-yl)methyl-formylamino]benzoyl]amino]pentanedioic acid), and PteGlu (pteroylglutamate (2*S*)-2-[[4-[(2-amino-4-oxo-3*H*-pteridin-6-yl)methylamino]benzoyl]amino]pentanedioic acid) (Hanson and Gregory, 2002). The structures of folate vitamers analyzed throughout this study are illustrated in Figure 1.

Since there is an increased interest in the use of microalgae as functional food, the quantitative analysis of folates in well-characterized strains would add significantly to knowledge about the nutritional composition and value of microalgae. At present, there is only scattered information about the total folate content of microalgae biomass. Two publications presented microbiological assays for quantification with limited analytical robustness (Brown et al., 1999; Fujii et al., 2010). Folate quantification by microbiological assays are sensitive, but the results are biased by choice of the calibrants and reagents (Ringling and Rychlik, 2017a). Recently, Edelmann et al. published reliable folate data on commercially available microalgae using microbiological assay and an UHPLC method (Edelmann et al., 2019). However, using UHPLC, particular attention to a complete deconjugation of polyglutamates to the respective monoglutamates need to be paid as only monoglutamates can be detected (Ringling and Rychlik, 2017a). For a direct, reliable, and sensitive analysis, we applied a mass spectrometry-based stable isotope dilution assay (SIDA), which has demonstrated its superiority in folate quantification (Rychlik, 2011). SIDA is based on the application of isotopologic labeled internal standards, which present almost identically chemical and physical properties. As pointed out above SIDA has key advantages such as a complete compensation for losses of analytes during extraction and for ion suppression during LC-MS/MS measurements. Folate analysis using SIDA enables differentiation of the folate pattern (Asam et al., 2009) as well as the detection of an incomplete deconjugation by LC-MS/MS (Ringling and Rychlik, 2017a).

This study evaluates the differential folate content of industrial processed microalgae biomass including fresh biomass and strains isolated from the environment. We examined the folate content of lab-grown microalgae, in particular also marine microalgae as these organisms display higher process robustness in extreme saline media, and can be cultivated on non-arable land using sea, brackish or wastewater (Schenk et al., 2008). Additionally, it prevents culture instability in open pond reactor systems as many terrestrial contaminants, such as bacteria and filamentous fungi, cannot thrive in this medium (Rothschild and Mancinelli, 2011). Therefore, the large scale halophilic algae cultivation potentially shows better economic and ecological benchmarks than its freshwater equivalents (Schenk et al., 2008; Apel and Weuster-Botz, 2015). At present, there is little literature evidence of the nutritional value, specifically focused on folate production and vitamers with respect to halophilic algae strains (Kay and Barton, 1991; Brown et al., 1999; Fujii et al., 2010; Wells et al., 2017). We hypothesize, that in general halophilic green microalgae contain higher excess of folates compared to terrestrial, folate containing food sources, such as rice, strawberries and liver. In this context, it is noteworthy that there is currently no systematic study that elucidates algae strains with a specific analytical procedure that can distinguish folate vitamer species. Moreover, it has not been elucidated yet if processed biomass retains high folate contents compared to its freshly harvested counterpart. Thus, we analyzed total folates contents in freshwater and marine microalgae by SIDA using LC-MS/MS and examined the effects of different industrially applied cultivation conditions on total folate content.

## Materials and Methods

### Commercial Grade Food Supplements

Biomass was sampled from algae food supplement products obtained from Terra Elements GmbH, Munich, Germany (*Chlorella vulgaris*) and Feelgood Shop BV, RK Venlo, Netherlands (*Chlorella vulgaris*). A single batch analysis was performed. The products were bought as dried powders and analyzed unprocessed.

### Cultured Reference Strains

Reference microalgae strains found in Table 1 were obtained from the Sammlung von Algenkulturen der Universität Göttingen (SAG) and the Culture Collection of Algae and Protozoa (CCAP). Sample strains were transferred and adapted under sterile culture conditions to growth media.

**Table 1:** Overview of analyzed microalgae biomass.

**Genus; species; strain**	**Origin [country, place (CC ID)]**	**GPS coordinates (DMS)**	**Medium**	**NaCl content (w/v)**
**COMMERCIAL GRADE FOOD SUPPLEMENTS**				
*Chlorella vulgari*	Terra Elements GmbH, Munich, Germany Feelgood Shop BV, Venlo, Netherlands	n.a.	n.a.	n.a.
*Chlorella vulgaris*		n.a.	n.a.	n.a.
**CULTURED REFERENCE STRAINS**				
*Chlorella vulgaris*	n.a. (SAG 211-12)	n.a.	BG11	0%
*Porphyridium purpureum*	UK, Brixham (CCAP 1380/3)	n.a.	ASW	3%
*Microchloropsis salina*	UK, Isle of Cumbrae (SAG 40.85)	N 055° 46.081′, W 004° 55.18464	BG11	3%
**CULTURED ISOLATED STRAINS**				
*Picochlorum* sp. isolate	Australia, Salt Creek	S 036° 09.6874′, E 139° 38.8328′	ASP-M	5%
*Dunaliella salina* isolate	Australia, Salt Creek	S 036° 09.6874′, E 139° 38.8328′	J/1	5%
*Tetradesmus* sp. isolate	Australia, Salt Creek	S 036° 09.6874′, E 139° 38.8328′	BG11	3%
*Chlorella* sp. isolate	Australia, Brisbane River	n.a.	BBM	0%

*n.a., not available; NaCl, Sodium chloride; CC ID, Culture collection identification; DMS, Degrees-Minutes-Seconds*.

### Cultured Isolated Strains

Isolated microalgae strains were enriched and purified from Australian environmental samples obtained in August 2014 by Thomas Brueck and coworkers under permission of the Australian government. Preparation of repeated serial dilution cultivations resulted in unialgal cultures after sampling. Maintained unialgal cultures were checked for purity by microscopy (Zeiss AxioLab, Carl Zeiss AG, Oberkochen, Germany) and in high throughput by fluorescence-activated cell sorting analysis (Biorad S3 Sorter, Bio-Rad Laboratories, Inc., Hercules, USA).

### Microalgae Cultivation

All reference and isolated microalgae samples were cultured in 500 ml Erlenmeyer flasks with a fill volume of 200 ml in New Brunswick Innova 44 series shakers (26°C, 120 rpm) fitted with light emitting diodes (Future LED GmbH, Berlin, Germany) (Figure S1). The cultures were inoculated with fresh cultivars at a starting optical density of 0.1 (OD_750_). A constant visible sunlight spectrum approximation illumination at a Photosynthetic Photon Flux Density (PPFD) of 200 μmol m^−2^ s^−1^ was applied. Individual aeration with 1% v/v CO_2_ enriched air was controlled by a DASGIP^®^ MX module (Eppendorf AG, Hamburg, Germany). A constant gas flow equivalent to 6 head volume exchanges per hour was applied. After 14 days of cultivation, the resulting algae biomass was harvested by centrifugation (2,450 × g, 5 min). No washing steps were applied. Subsequently, the frozen pellet (−80°C) *Chlorella* sp. isolate was freeze-dried for 24 h. Dried samples were sealed under nitrogen-enriched air, subdued light and stored at −80°C until further processing. Moreover, the analysis of folate content and vitamer distribution after 14 days of cultivation and subsequent 24-h osmotic stress, nitrogen limitation and light spectrum shift was studied. A batch of 15 Erlenmeyer flask cultures where combined after 14 days. The homogeneous culture was pelleted by centrifugation (2,450 × g, 5 min) in individual batches and re-suspended in BBM medium for the control, green light and blue/pink light groups in triplicates. Nitrogen limited conditions were initiated by resuspension in NaNO_3_ free BBM media. Osmotic stress was initiated by resuspension in 1% NaCl containing BBM media. After 24 h the cultures were harvested and stored for folate analysis as described above. LED spectrum is shown in Figure S2.

All cultivation media were prepared sterile. The microalgae media BG11 [Blue-Green Medium] (Allen, 1968), J/1 [Johnson's media] (Allen, 1968), ASW [Artificial Seawater] (Allen and Nelson, 1910), ASP-M [Artificial Seawater medium by Provasoli] (Provasoli et al., 1957) and BBM [Bold's Basal Medium] (Bischoff and Bold, 1963) were prepared as published, but without organic nutrients, vitamins or complex components; all media recipes where adapted accordingly and obtained from: BG11 and J/1 (Borowitzka and Borowitzka, 1988); BBM, ASW and ASP-M (Anderson, 2005). The salinities were adjusted by the addition of sodium chloride as listed in Table 1.

### Phylogenetic and Fatty Acid Profile Characterization of Analyzed Biomass

Genomic DNA extracts were prepared using the InnuPrep plant DNA extraction kit (Analytic Jena AG, Jena, Germany, 845-KS-1060050). 18S rDNA amplification by PCR (Eppendorf AG, Germany, Mastercycler nexus) was conducted using the primers EukA (21F) (5′- AACCTGGTTGATCCTGCCAGT-3′) (Medlin et al., 1988) and EukB (1791R) (5′-TGATCCTTCTGCAGGTTCACCTAC-3′) (Medlin et al., 1988). The purified amplicons were sequenced by capillary sequencing (Eurofins Genomics GmbH, Ebersberg, Germany). The reads were searched against the GenBank database by the BLASTn (Altschul et al., 1990) algorithm. The nearest ancestral microalgae sequence hits of isolates were used for the lineage assignments. The phylogenetic tree was built with Geneious Tree Builder (Biomatters Ltd., New Zealand, Geneious software version 11.1.3). 18S rDNA data was included from deposited reference strains which are obtainable from SAG, UTEX, CCAP.

The Fatty Acid Methyl Esters (FAME) were prepared according to reported methods applied to microalgae biomass with the following modification (Griffiths et al., 2010): 10 mg lyophilized biomass of each sample was used; the internal standard C17-TAG was replacement by C19-TAG Sigma Aldrich T4632 (Merck AG, Darmstadt, Germany). Initial extraction with GC grade toluene was assisted by sonification for 40 min in an ice bath. BF_3_ methanol was replacement by a HCl/Methanol Supleco 17935 solution (Merck AG, Darmstadt, Germany). After transesterification FAMEs were extracted with GC grade hexane by vortexing with glass beads for 10 s at RT. After centrifugation for 5 min at 1,000 rpm, the hexane phase was transferred to GC-vails. The fatty acid profiles were measured with a Shimadzu GC-2025 equipped with a AOC-20i autosampler and FID detector (245°C) (Shimadzu, Kyoto, Japan). For each sample, 1 μl was injected with a split ratio of 1:10 at a temperature of 240°C and loaded on a Zebron ZB-WAX column (30 m × 0.32 mm, thickness of 0.32 μm, Phenomenex, Torrance, USA). The column oven temperature was set to 150°C for 1 min, a ramp of 5°C/min increased the temperature to a final target of 240°C. Thereafter holding the final temperature for 6 min. Hydrogen 5.0 was used as carrier gas at a constant flow rate of 35 ml/min. Identification of peaks was done by manual calibration with the external standard Marine oil FAME Mix (RestekGmbH, Bad Homburg, Germany). A relative quantification was done by integral FID signal comparison after normalization and exclusion of the internal C19 Fame Standard.

### Folate Content and Isoform Distribution Analysis on Microalgae Biomass

For the folate stable isotope dilution assay analysis, the complete information about chemicals, standards, and preparations of solutions as well as validation of the method can be obtained from a previous publication (Striegel et al., 2018b). Briefly, LODs of all analytes were in the range of 0.17 and 0.33 μg/100 g, and LOQs in the range of 0.51 and 0.96 μg/100 g. Inter-injection precision was between 1.96 and 4.46%, intra-day precision between 2.44 and 4.60%, and inter-day precision between 3.04 and 5.06%. 10-CHO-PteGlu was quantified using [^13^C_5_]-5-CHO-H_4_folate as internal standard. The LODs and LOQs were estimated using the response factor of 5-CHO-H_4_folate as a reference value. We calculated a LOD of 0.14 μg/100 g and a LOQ of 0.40 μg/100 g. All results given in the results and discussion section are based on dry biomass.

### Sample Preparation for the Folate Analysis of Algae Biomass

The sample extraction was performed under subdued light. Briefly, the freeze-dried algae samples were finely grounded using a mortar and pestle. Ten milligram of resulted biomass were used for each extraction. After addition of 10 ml buffer (200 nmol/l 2-(N-morpholino)ethanlsulfonic acid hydrate (MES), 114 nmol/l ascorbic acid, 0.7 nmol/l DTT, pH 5.0) internal standards ([^13^C_5_]-PteGlu, [^13^C_5_]-H_4_folate, [^13^C_5_]-5-CH_3_-H_4_folate, and [^13^C_5_]-5-CHO-H_4_folate [used for quantitation of 5-CHO-H_4_folate and 10-CHO-PteGlu)] were added to samples in equal amounts (0.004–0.5 nmol) to the anticipated respective analyte content. For deconjugation, an adjusted amount of 1 ml rat serum (used without dilution; endopeptidase used for deconjugation of diglutamates to monoglutamates) and 2 ml chicken pancreas suspension (1 g/l in phosphate buffer (100 mmol/l, 1 g/l ascorbic acid, pH 7); exopeptidase used for deconjugation of polyglutamates to the respective diglutamates) were added, and the samples were incubated overnight for a minimum of 12 h in a water bath at 37°C. After a 10 min boiling step, samples were cooled on ice and transferred into plastic centrifuge tubes with additional 10 ml acetonitrile. After centrifugation (20 min, 4,000 rpm, 4°C), the extracts were applied to a solid-phase extraction (SPE) clean-up using strong anion-exchange (SAX) cartridges (quaternary amine, 500 mg, 3 ml). In short, cartridges were activated with two volumes of methanol, equilibrated with 3 volumes of buffer [10 mmol/l phosphate buffer (consisting of 100 mmol/l sodium hydrogen phosphate and adjusting the solution with 100 mmol/l dipotassium hydrogen phosphate to pH 7.0) was mixed with 1.3 mmol/l DTT], extracts were completely applied, and cartridges were washed again with 3 volumes of buffer for equilibration and run dry. The folates were eluted using 2 ml of buffer for elution (5% sodium chloride, 1% ascorbic acid, 100 mmol/l sodium acetetate, and 0.7 mmol/l DTT), membrane filtered, and measured by LC-MS/MS.

### LC-MS/MS

Chromatography was carried out on a Shimadzu Nexera X2 UHPLC system (Shimadzu, Kyoto, Japan) with a Raptor ARC-18 column (2.7 μm, 100 × 2.1 mm, Restek, Bad Homburg, Germany) and a Raptor ARC-18 precolumn (2.7 μm, 5 × 2.1 mm, Restek, Bad Homburg, Germany) as a stationary phase that was kept at 30°C. The mobile phase consisted of (A) 0.1% formic acid and (B) acetonitrile with 0.1% formic acid delivered as a binary gradient at a flow rate of 0.4 ml/min. Gradient concentration started at 3% B and raised linearly to 10% within the next 2.5 min and held at 10% for further 2.5 min. Then, the concentration went up to 15% B within 5 min and then to 50% within 1 min, followed by holding at 50% B for 1 min. Within 1 min, the concentration returned to 3% B and was equilibrated for 4 min. The injection volume was 10 μl.

The LC was interfaced with a triple quadrupole mass spectrometer (LCMS-8050, Shimadzu, Kyoto, Japan). It was operated in the positive ESI mode for all analytes. The specified settings are also described previously (Striegel et al., 2018b). Data acquisition was performed with LabSolutions software 5.8 (Shimadzu, Kyoto, Japan). The ion source paramaters were set as follows: heat block (400°C), dilution line (250°C), interface temperature (300°C), drying gas (10 l/min), heating gas (10 l/min), nebulizing gas (3 l/min), collision-induced dissociation gas (270 kPa), and interface voltage (4 kV), respectively. MS parameters were listed in the previously published paper about the method validation. The mass spectrometer was operated in the multiple reaction monitoring (MRM) mode for MS/MS measurements.

### Statistical Evaluation

All folate results are means of technical triplicates ± standard deviation. The received values were tested for normal distribution with the test of Kolmogorov-Smirnov and were tested for outliers by the test of Dixon. A significance test was carried out by the *T*-test after applying the F-test for heteroscedasticity. The level of statistical significance was set to *p* < 0.05.

## Results

**Figure 2:**
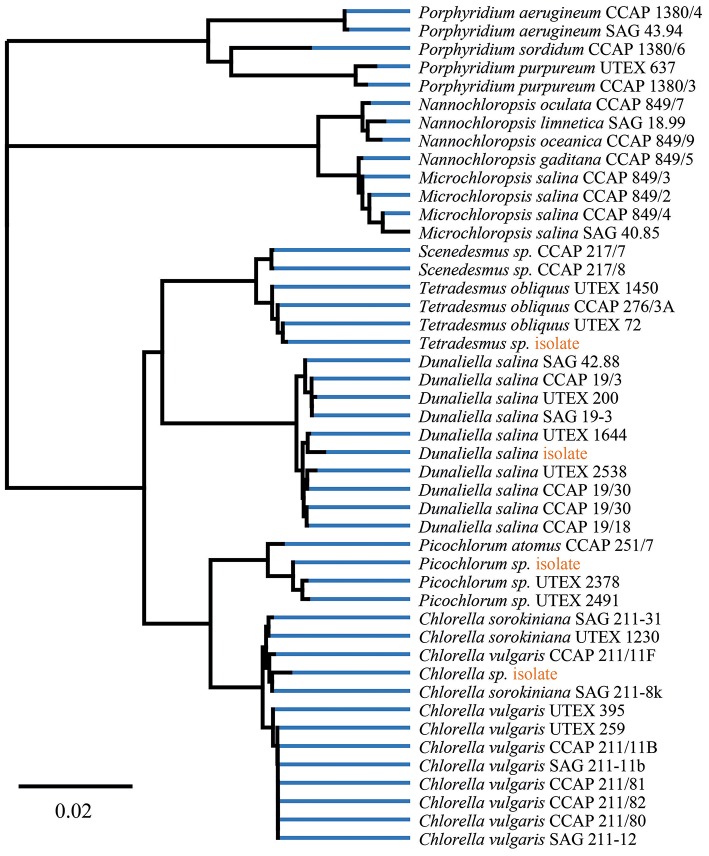
Phylogenetic tree of isolates and reference strains 18S rDNA data build by Geneious Tree Builder [Geneious 11.1.3 (https://www.geneious.com)]. Visualization of global alignment and Neighbor-Joining with 93% similarity cost matrix using the Tamura-Nei distance model.

Within this project, a series of freshwater and marine microalgae were analyzed for their total folate content and vitamer distribution by SIDA based on dry biomass. For all cultured microalgae strains, we validated the lineage assignment by sample-specific fatty acid profiles and 18S rDNA sequence identity. To reflect on the analyzed diversity, we included a phylogenetic tree with reference strains from UTEX, SAG and CCAP collections. The construction of the phylogenetic tree (Figure 2) indicates a clustering according to the samples previous lineage assignments or reference. The 18S rDNA sequences of isolated microalgae strains are deposited in the NCBI database, and the accession numbers are listed in Table 1. The reference and environmental microalgae strains cluster with the reference Chlorophyta strains, nearest relatives obtainable from popular culture collections can be deduced from the phylogenetic tree shown in Figure 2. For enhanced strain validation and culture status, we determined the fatty acid profile (Table 2). All isolated and deposited microalgae samples showed similar fatty acid profiles as reference data (Lang et al., 2011). The red algae *P. purpureum* showed a previously observed 41.9% eicosatetraenoic acid content in total transesterified lipid extracts.

**Table 2:** Fatty acid composition of microalgae samples by GC-FID analysis.

**Fatty acid methyl esters (FAME) [%]**	**C16:0**	**C16:1**	**C18:0**	**C18:1**	**C18:1**	**C18:2**	**C18:3**	**C20:4**	**C20:5**	**18S rDNA accession number**
**CULTURED REFERENCE STRAINS**
*Chlorella vulgaris SAG 211-12*	24.2	9.1	Trace	5.7	Trace	25.1	34.6	ND	ND	MK971791
*Porphyridium purpureum CCAP 1380/3*	26.8	Trace	Trace	2.6	Trace	16.8	ND	41.9	8.9	MK971789
*Microchloropsis salina SAG 40.85*	34.1	5.7	Trace	8.7	Trace	40.7	7.6	ND	ND	MK971790
**ISOLATED STRAINS**
*Picochlorum* sp. isolate	35.9	Trace	1.7	16.8	Trace	23.1	19.5	ND	ND	MK973100
*Dunaliella salina* isolate	23.0	9.3	1.0	7.8	1.6	25.3	30.1	ND	ND	MK973098
*Tetradesmus* sp. isolate	31.4	1.0	Trace	26.1	1.1	23.0	13.2	ND	ND	MK973099
*Chlorella* sp. isolate	24.3	Trace	Trace	7.2	1.8	23.4	31.4	ND	ND	MN365023

*ND, not detected; Trace <1%*.

**Figure 3:**
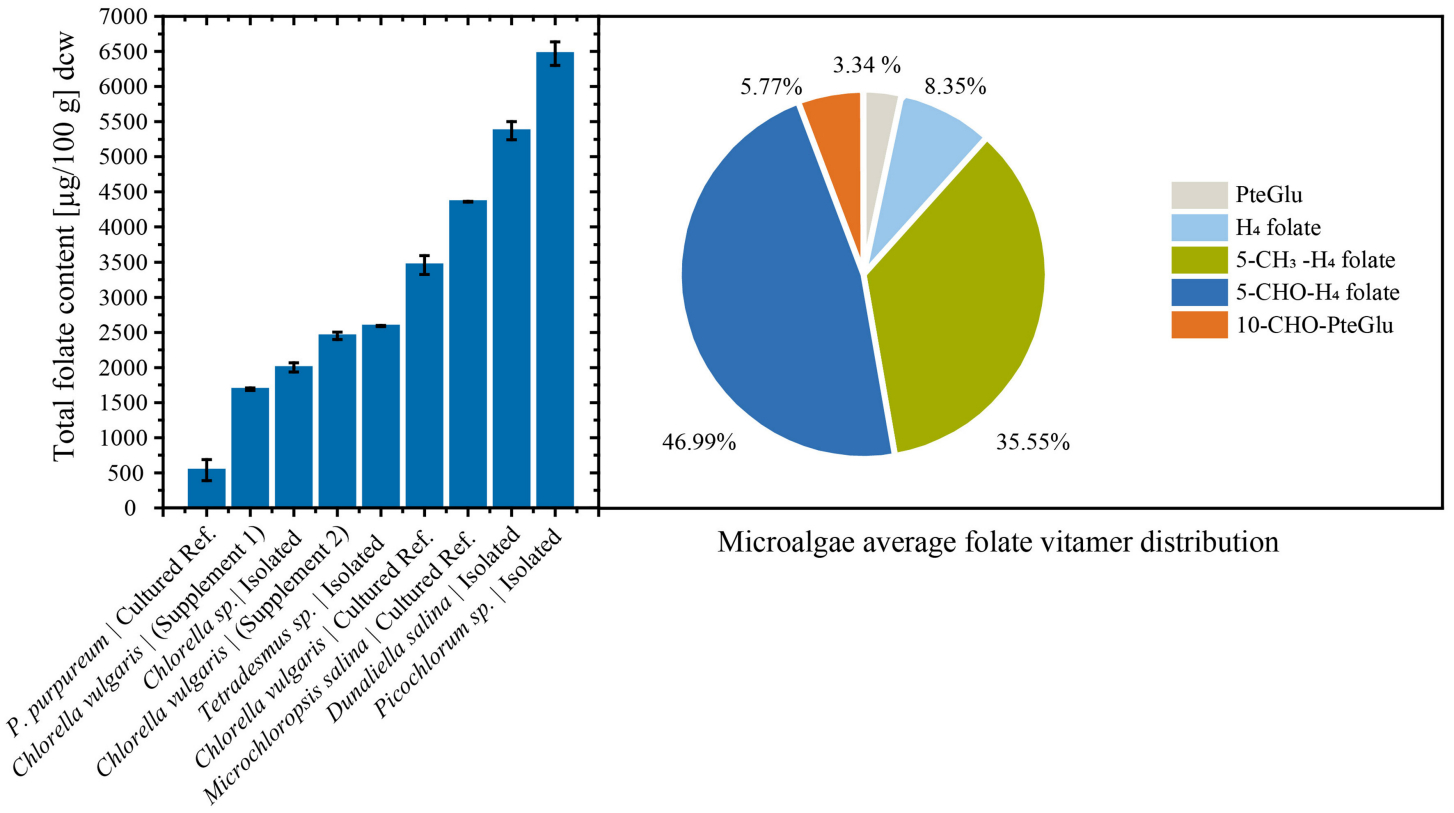
**(Left)** Total folate content of food supplements, fresh cultured biomass, and isolated strains normalized by dry cell weight (dcw) biomass. **(Right)** Average folate vitamer distribution of the analyzed microalgae.

Consistently, high folate contents were detected in all algae biomasses samples, in the range between 539 ± 150 μg/100 g and 6,470 ± 167 μg/100 g. The commercial reference food supplements showed high folate contents of 1,690 ± 17.3 μg/100 g (Supplement 1) and 2,450 ± 52.1 μg/100 g (Supplement 2), respectively. For the cultured reference *Chlorella vulgaris (SAG211-12)* biomass a folate content of 3,460 ± 134 μg/100 g was detected. The highest overall total folate content was observed in *Picochlorum* sp. isolate with a very high value of 6,470 ± 167 μg/100 g in dry biomass thus revealing a significantly higher [*p* < 0.05, *n* = 1 (biological)] total folate content compared to all other samples. The lowest total folate content was observed in *P. purpureum* biomass with a value of 539 ± 150 μg/100 g. The folate contents analyzed in microalgae are graphically shown in Figure 3 (left), and the detailed values are listed in Table 3.

**Table 3:** The folate content and vitamer distribution of selected microalgae strains, calculated as PteGlu in [μg/100 g].

**Genus; species; strain**	**5-CH_**3**_-H_**4**_folate**	**5-CHO-H_**4**_folate**	**10-CHO-PteGlu**	**H_**4**_folate**	**PteGlu**	**Total folate content**
**COMMERCIAL FOOD SUPPLEMENTS**						
*Chlorella vulgaris* (Supplement 1)	397 ± 11.6	1010 ± 17.3	119 ± 1.81	60.6 ± 6.41	108 ± 7.86	1690 ± 17.3
*Chlorella vulgaris* (Supplement 2)	631 ± 82.7	1510 ± 65.8	132 ± 7.58	102 ± 17.9	73.3 ± 2.53	2450 ± 52.1
**CULTURED REFERENCE STRAINS**						
*Chlorella vulgaris SAG 211-12*	2420 ± 47.8	643 ± 4.29	168 ± 83.9	216 ± 13.6	7.96 ± 3.88	3460 ± 134
*Porphyridium purpureum CCAP 1380/3*	191 ± 40.3	137 ± 28.4	67.7 ± 75.0	71.0 ± 28.4	71.5 ± 4.74	539 ± 150
*Microchloropsis salina SAG 40.85*	3050 ± 50.0	1060 ± 50.0	77.2 ± 4.55	147 ± 2.50	23.2 ± 1.45	4360 ± 5.60
**CULTURED ISOLATED STRAINS**						
*Picochlorum* sp. isolate	4020 ± 8.87	1990 ± 124	76.9 ± 10.8	285 ± 73.0	96.4 ± 22.1	6470 ± 167
*Dunaliella salina* isolate	847 ± 33.7	3960 ± 48.0	255 ± 28.6	269 ± 43.3	39.8 ± 23.5	5370 ± 129
*Tetradesmus* sp. isolate	1410 ± 8.62	731 ± 14.5	82.3 ± 1.48	306 ± 4.54	67.0 ± 0.68	2590 ± 9.59
*Chlorella* sp. isolate	718 ± 99.9	832 ± 82.3	38.6 ± 4.93	407 ± 40.3	1.40 ± 0.70	2000 ± 66.6

Moreover, we differentially analyzed the main vitamers present in food encompassing PteGlu, H_4_folate, 5-CH_3_-H_4_folate, 5-CHO-H_4_folate, and 10-CHO-PteGlu. The average vitamer distribution is shown in Figure 3 (right), and a detailed distribution is listed in Table 3. The main vitamers were 5-CH_3_-H_4_folate (5.43–70.1%) and 5-CHO-H_4_folate (18.6–80.9%). The minor vitamers were 10-CHO-PteGlu (1.19–12.6%), H_4_folate (0.54–21.2%), and the fully oxidized PteGlu (0.07–13.3%). 5-CH_3_-H_4_folate was the main vitamer in *Tetradesmus* sp. isolate, *Microchloropsis salina SAG 40.85, Chlorella vulgaris SAG 211-12*, and the *Picochlorum* sp. isolate.

**Figure 4:**
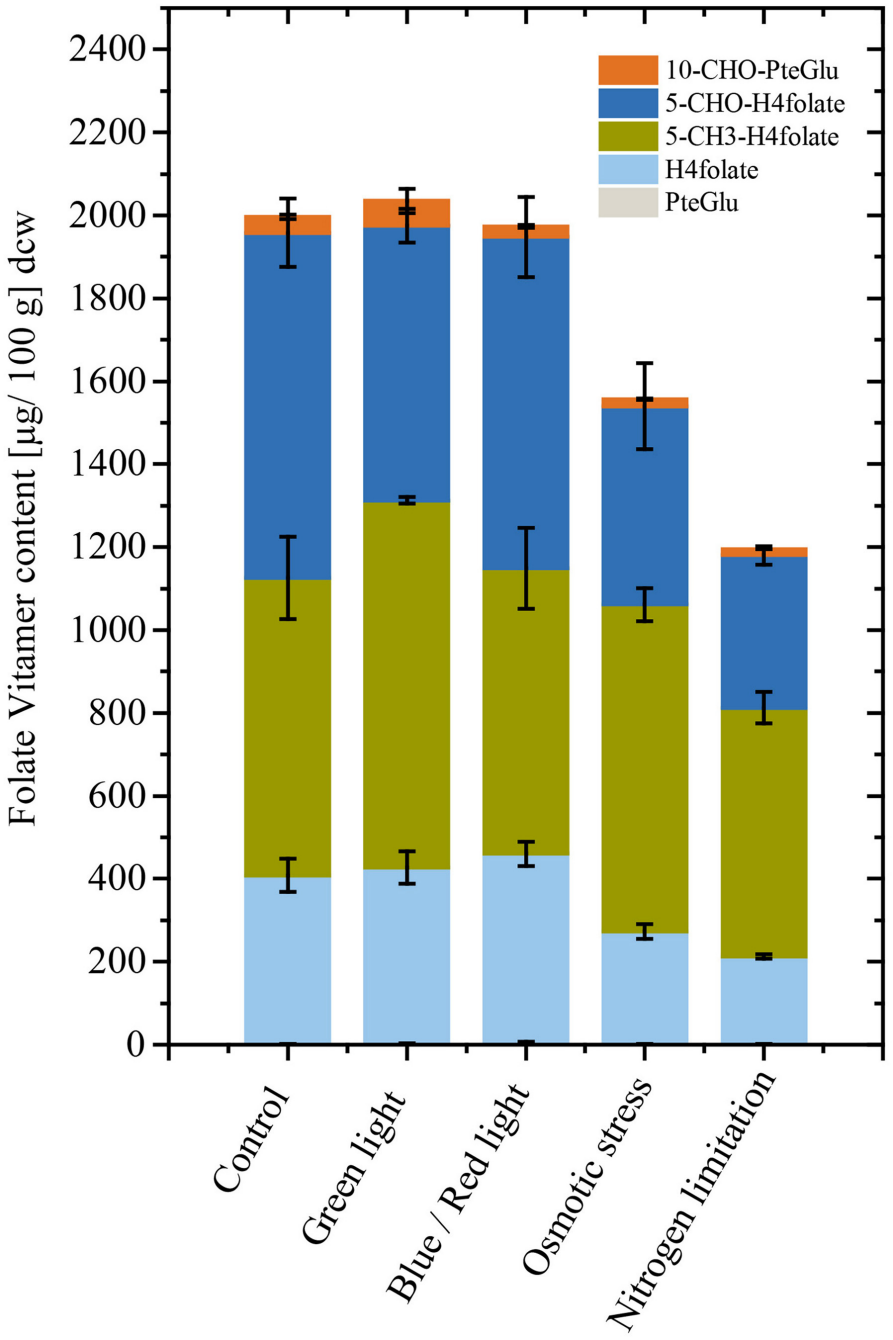
Total folate content and vitamer distribution after conditional changes applied for 24 h in triplicates. *Chlorella* sp. isolate cultivations show reduced folate content when exhibited to osmotic stress and nitrogen limitation. Light wavelength changes did not affect folate contents when shifted for 24 h.

Microalgae cultivation often requires product accumulation measures to increase obtainable yields in a technical scale. Particularly, effects of osmotic stress in case of pigment production and nitrogen limitation in case of lipid production are utilized (Borowitzka et al., 1990; Wang et al., 2019). The model microalgae *Chlorella* sp. isolate was used in an additional experiment to evaluate effects of widely applied stressors during late cultivation stages. Osmotic stress for 24 h did have a significant negative effect [*p* < 0.05, *n* = 3 (biological)] on total folates in *Chlorella* sp. isolate (Figure 4). Also, nitrogen limitation resulted in reduced [*p* < 0.05, *n* = 3 (biological)] detectable total folate contents. In contrast, light spectrum shift did not show differences on total folate. The detailed values and illumination conditions are shown in Table S1.

## Discussion

In this study, an analysis of the total folate content of a genetically defined microalgae series with a focus on halophilic Chlorophyta has been carried out. We measured very high total folate contents in all analyzed microalgae samples. The cumulative data indicate that microalgae can serve as a concentrated source of natural folates in form of food supplement products for folate-deficient populations. However, the set of analyzed microalgae species showed high variations in total folate, giving room for process and strain optimization toward concentrated algae food products. As a reference to our cultivated algae species, we measured the folate content of commercial, algae food supplement products based on *Chlorella* biomass. Interestingly, the industrially processed dried *Chlorella* biomass retained a relatively high folate content, which however was only half of the total amount detected in cultured *Chlorella vulgaris*. *Chlorella* biomass has gained industrial attention in the past due to their generally regarded as safe (GRAS) status declared by the U.S. Food and Drug Administration (FDA). This GRAS status renders novel food approval of these products unnecessary and allows a rapid market rollout of these products. While our isolated halophilic algae strains *Dunaliella* sp. and *Picochlorum* sp. displayed significantly higher folate yields than *Chlorella*, the regulatory barriers for novel food would delay their market entry despite their excellent nutritional content. What is more, the biomass of the *Picochlorum* sp. isolate showed a total folate content of 6,470 μg/100 g, which is currently the highest total folate content detected in algae. This results further emphasizes the importance of halophilic microalgae as an excellent source of micronutrients. Previous publications reported total folate contents within the same order of magnitude as our results. Brown et al. analyzed the vitamin content of four Australian microalgae, inter alia *Nannochloropsis* sp., *Pavlova pinguis, Stichococcus* sp., and *Tetraselmis* sp. (Brown et al., 1999). The latter authors detected folate concentrations ranging between 1,700 and 2,600 μg/100 g. Microalgae collected in Japanese ponds were found to contain total folate contents in a range between 1,500 and 3,600 μg/100 g in dry biomass (Fujii et al., 2010). As previous studies applied microbiological assays to determine the folate content, they were not able to determine the folate pattern. Recently, Edelmann et al. analyzed various commercially available microalgae powder using microbiologial assays and an UHPLC methods to differentiate between different vitamers (Edelmann et al., 2019). The group found in average significantly higher total folate contents in *Chlorella* (2,500–4,700 μg/100 g) and *Nannochloropsis gaditana* (2,080 μg/100 g) compared to *Spirulina* (250–470 μg/100 g). The reviewed amounts of *Chlorella* agree with the folate contents in this study.

Furthermore, we found 5-CH_3_-H_4_folate and 5-CHO-H_4_folate as the main vitamers in all algae isolates studied, which is in accordance with Edelmann et al. (2019). The latter authors also quantified 5,10-CH^+^-H_4_folate and found not negligible amounts of this vitamer in Spirulina samples. This vitamer should be included in future analysis of microalgae. As previous studies applied microbiological assays or UHPLC methods to determine the folate content, this is the first report of the folate vitamer distribution in microalgae by high-resolution mass spectrometry. As folate vitamers show different stabilities and conversion reactions, the folate pattern can give additional information about the stability of folates in microalgae.

The absorption capacity, as well as the post-absorptive metabolism of different vitamers, lacks deeper understanding (Visentin et al., 2014). However, due to the different stability of folate vitamers, we assume that H_4_folate is less bioavailable (Ringling and Rychlik, 2017b). Furthermore, to shed light on the extend of absorption of folates from algae, human trials are required. In the context of species-specific folate concentrations our results for the *Dunaliella* genus exceeded previously reported values (*Dunaliella tertiolecta*, 480 μg/100 g dry biomass) by the factor of ten. In this context it remains to be demonstrated, whether this discrepancy is due to physiological and phenotypic states of the algae or based on subspecies genetic variations.

High folate and vitamin concentration in microalgae biomass is not a generality (Croft et al., 2006); and we observed significantly lower folate content in the studied red algae *P. purpureum*. Osmotic stress and nitrogen limitation during cultivation showed negative effects on total folate content in *Chlorella* sp. As far as we know, this is the first report of precise stress effects during microalgae cultivations, indicating that nitrogen starvation and increased salinity impacts on total folate content negatively in *Chlorella* sp. This is particularly interesting for cultivation processes of microalgae biomass for food and feed. Typical valorization concepts for marine algae biomass includes multiple product streams, and relies on lipid and pigment accumulation, which seems at least in the model algae *Chlorella* sp. to be unfavorable for folate production. Also, *Dunaliella* sp. isolates showed a reduced total folate content when samples were analyzed from the stationary cultures (data not shown). However, only limited and contradictory results on folate accumulation under differential growth conditions are documented (Brown et al., 1999). Folate synthesis is associated with assimilation processes, thus we postulate that highest folate concentrations will be found under unlimited growth conditions (Hjortmo et al., 2008). Further studies are needed to shed light on the underlying metabolic regulation of folates in freshwater and marine microalgae. Product stability is a major concern as degradation of folates is known (Fitzpatrick et al., 2012; Blancquaert et al., 2015) rendering supplement product formulation, packaging, and distribution similar decisive as developments in process and strain optimization (Blancquaert et al., 2015).

**Figure 5:**
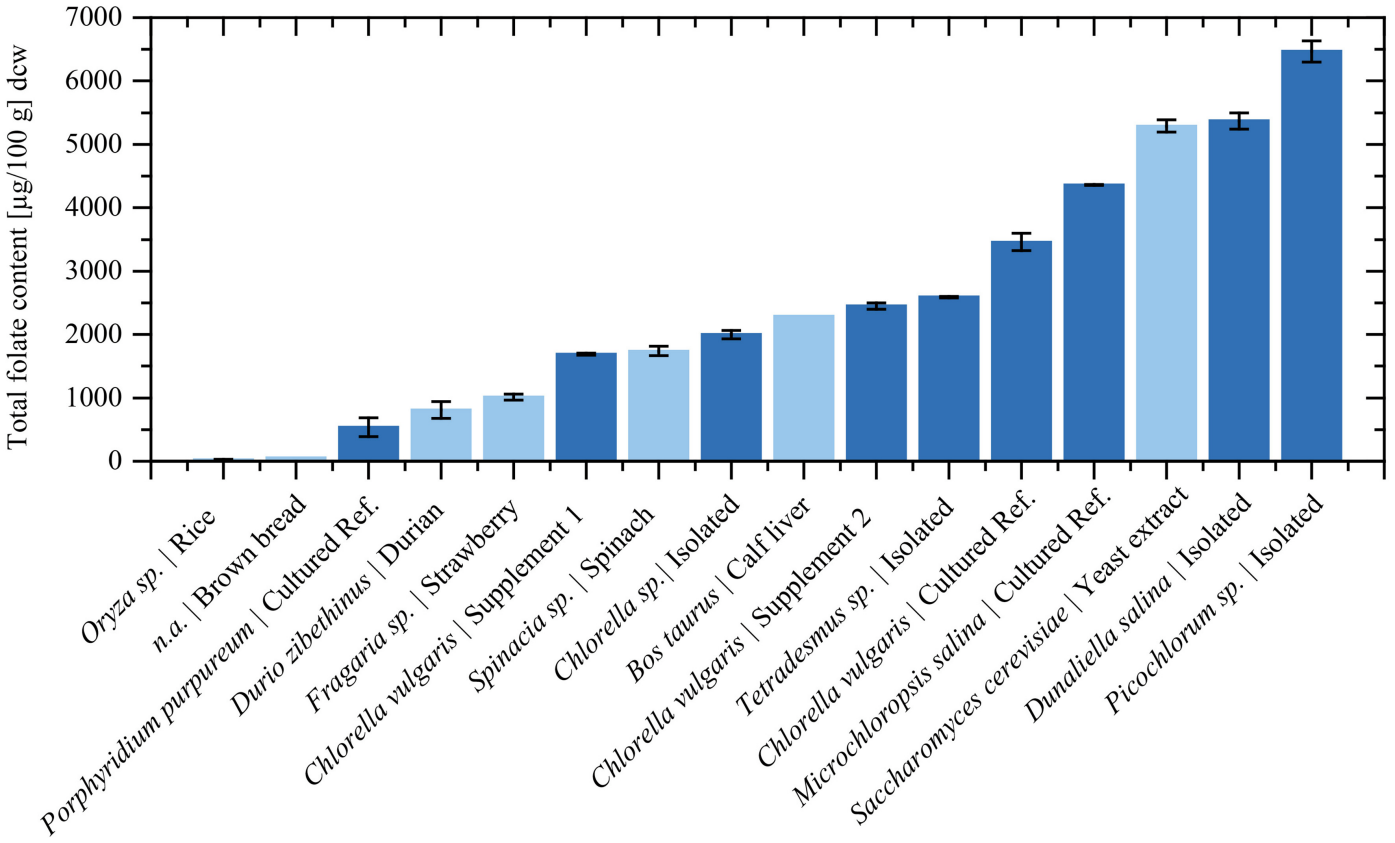
The total folate content in selected food and algae dry biomass. Dark blue bars represent measured values from this study. The total folate content of food products was taken from previous publications using the same quantification method. The total folate contents were adapted to dry biomass according to literature values (Souci et al., 2008): Rice (*Oryza* sp.) from Ringling and Rychlik (2017a), Strawberry (*Fragaria* sp.) from Striegel et al. (2018b), Brown bread from Ringling and Rychlik (2013), Spinach (*Spinacia* sp.) (Ringling and Rychlik, 2013), Durian (*Durio zibethinus*) from Striegel et al. (2018a), Calf liver from Ringling and Rychlik (2017a), Yeast extract from Jacob et al. (2019).

**Figure 6:**
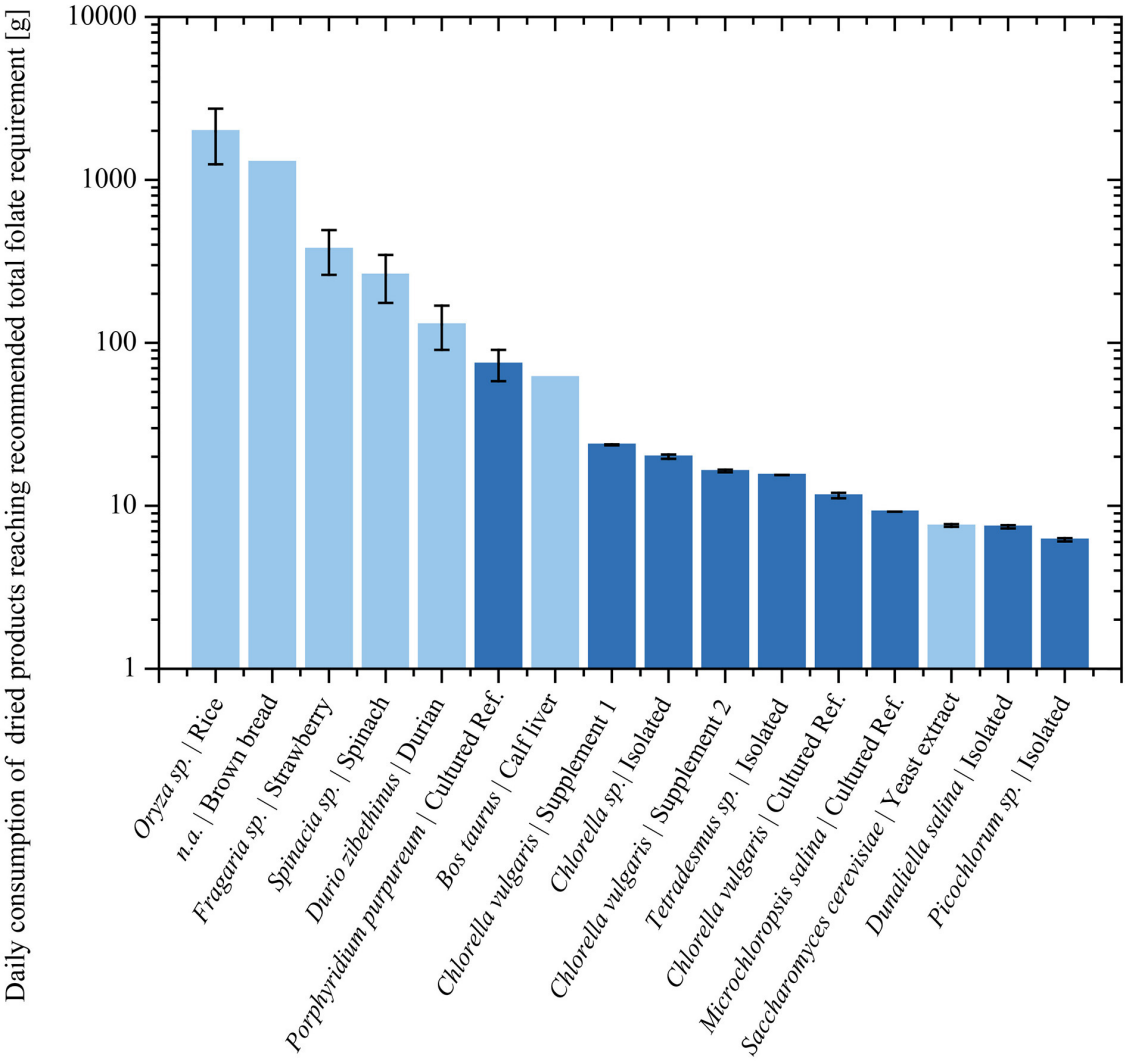
Analyzed food products during this (Dark blue) and previous (Light blue) studies. Required biomass consumption in logarithmic scale according to the U.S. RDA for folate intake.

Figure 5 depicts a summary of previously analyzed foods and supplements, presented in comparison with our results. In order to reach the European RDA of 300 μg or United States RDA of 400 μg (Krawinkel et al., 2014), an intake of only 6 g *Picochlorum* sp. dry biomass would be required. In contrast, reaching the RDA with high folate content fruits as strawberries would require about 400 g dried biomass (Figure 6). Thus, it is viable to flag algae as concentrated food and nutrient producing platforms. To the best of our knowledge, we are the first to report the folate content of different microalgae strains using SIDA as quantification method. Microalgae, the potentially highest source of folates, warrant further studies on the distribution of pteroylpolyglutamates as well as on bioaccessibility, absorption, and retention for physiological functions.

## Data Availability Statement

Generated sequencing reads can be found at NCBI database: MK971789, MK971790, MK971791, MK973100, MK973098, MK973099, MN365023.

## Author Contributions

LS, TF, and DW conceived the experiment, analyzed the data, and co-wrote the manuscript. LS, TF, DW, and NW performed experiments. MF reviewed writing. TB and MR supervised the project and reviewed writing.

### Conflict of Interest

The authors declare that the research was conducted in the absence of any commercial or financial relationships that could be construed as a potential conflict of interest.
