# Supplementary material for: Microalgae a Superior Source of Folates: Quantification of Folates in Halophile Microalgae by Stable Isotope Dilution Assay
Source: Front Bioeng Biotechnol. 2020 Jan 21;7:481. doi: 10.3389/fbioe.2019.00481 (PMC6985443; doi:10.3389/fbioe.2019.00481)
Supplement: Supplementary file 1 [file Data_Sheet_1.pdf]

Supplementary Material

Microalgae a Superior Source of Folates: Quantification of Folates in Halophile Microalgae by Stable Isotope Dilution Assay

Dirk Volker Woortman [1†], Tobias Fuchs [1†], Lisa Striegel [2†], Monika Fuchs[1], Nadine Weber[2], Thomas B. Brück [1] and Michael Rychlik [2,3]

[1] Werner Siemens-Chair of Synthetic Biotechnology, Technical University of Munich, Garching, Germany,
[2] Chair of Analytical Food Chemistry, Technical University of Munich, Freising, Germany,
[3] Centre for Nutrition and Food Sciences, Queensland Alliance for Agriculture and Food Innovation, The University of Queensland, Brisbane, QLD, Australia

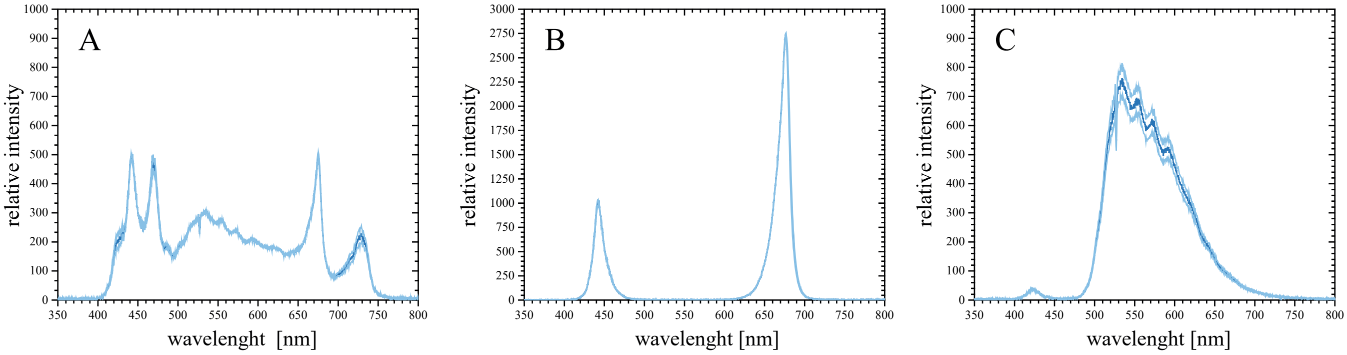

Figure S1: Wavelength spectra of cultivation system installed in a New Brunswick incubator (see Figure S2). A: Full visible spectrum applied to all cultivations during this study. B: Blue and red light spectrum as applied for 24h to the *Chlorella sp.* isolate stress experiment. C: Green light spectrum as applied for 24h as stressor. Spectra recorded with a STS-VIS spectrometer. (OceanOptics, Inc., Largo, USA)

Supplementary Material

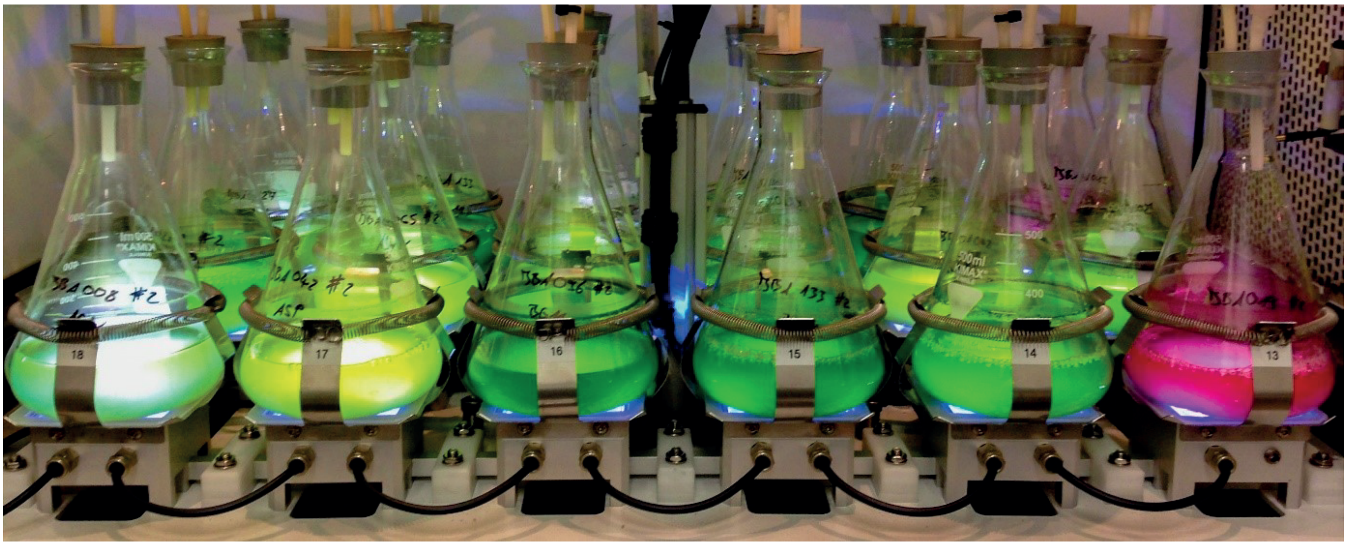

Figure S2: Image of the 500 ml Erlenmeyer flask cultivation system used for all experiments. Custom modification of New Brunswick Innova 44 series (Eppendorf AG, Hamburg, Germany) by Future LED GmbH (Berlin, Germany). Water cooled bottom illumination equipped with SunLike LEDs. Single point Air/CO_2 aeration by DasGip® MX module (Eppendorf AG, Hamburg, Germany).

Supplementary Material

Table S1:Total folate content and vitamer distribution in [µg/100 g] of *Chlorella sp.* isolate cultivations after conditional changes applied for 24 h in triplicates. *Chlorella sp.* isolate show reduced folate content when exhibited to osmotic stress and nitrogen limitation. Light wavelength changes did not affect folate content when shifted for 24 h.

Genus; species; strain	5-CH_3-H_4folate	5-CHO-H_4folate	10-CHO-PteGlu	H_4folate	PteGlu	Total folate content
Chlorella sp. isolate						
**Control (A)**	718 ± 99.9	832 ± 82.8	38.6 ± 4.93	407 ± 40.3	1.40 ± 0.70	2000 ± 66.6
**Blue / Red light (B)**	689 ± 97.8	799 ± 96.9	25.1 ± 2.99	456 ± 28.8	4.11 ± 3.44	1970 ± 67.2
**Green light (C)**	886 ± 8.36	663 ± 40.9	59.1 ± 28.6	425 ± 38.7	2.26 ± 2.11	2040 ± 10.7
**Osmotic stress**	789 ± 39.7	478 ± 103	17.2 ± 1.60	271 ± 17.6	1.16 ± 0.40	1560 ± 50.7
**Nitrogen limitation**	600 ± 38.2	368 ± 22.3	14.9 ± 0.51	211 ± 4.61	1.46 ± 0.99	1200 ± 28.7
